# P61 The role of chest imaging in antimicrobial stewardship: a systematic review of ultrasound and radiography in differentiating bacterial and viral pneumonia

**DOI:** 10.1093/jacamr/dlaf230.068

**Published:** 2025-12-04

**Authors:** Waleed Mostafa Marzok, Rasha Abdelsalam Elshenawy

**Affiliations:** Physiological Measurement Ltd, Oswestry, SY11 1AJ UK; Department of Medicine, School of Health, Medicine and Life Sciences, University of Hertfordshire, Hatfield, UK

## Abstract

**Background:**

Community-acquired pneumonia (CAP) in elderly patients remains a leading cause of morbidity and mortality, and inappropriate antibiotic use contributes significantly to antimicrobial resistance (AMR).^1^ Imaging plays a pivotal role in the diagnostic pathway, particularly chest radiography and ultrasound, which can help distinguish bacterial from viral pneumonia and guide targeted therapy.^2^ Incorporating imaging findings into antimicrobial stewardship (AMS) programmes could reduce unnecessary broad-spectrum antibiotic use, optimize treatment duration and improve patient outcomes.^3^ This systematic review aims to explore the role of chest imaging in AMS, focusing on elderly patients and those with comorbidities and renal impairment.

**Methods:**

A systematic literature search was conducted in PubMed, Scopus, Google Scholar and Cochrane Library (2015–2025) following PRISMA guidelines. Search terms included *antimicrobial stewardship*, *pneumonia*, *chest radiography*, *lung ultrasound*, *diagnostic imaging*, *elderly* and *renal impairment*. Studies were included if they: (i) evaluated the role of chest X-ray or ultrasound in CAP diagnosis; (ii) reported impact on antibiotic prescribing, dose optimization, or stewardship outcomes; and (iii) included elderly populations. Data analysis was conducted using qualitative and quantitative methods. Quality appraisal was performed using CASP and MMAT tools.

**Results:**

From the 2160 records screened, 12 studies met the inclusion criteria. Evidence indicated that chest ultrasound demonstrated sensitivity equal to or greater than chest radiography in diagnosing pneumonia, especially in elderly patients with multiple comorbidities. Several studies highlighted that incorporating imaging results into clinical decision-making pathways reduced unnecessary antibiotic initiation (by 18–32%) and facilitated earlier IV-to-oral switching. Imaging-informed antimicrobial stewardship (AMS) strategies also improved renal dose adjustments in patients with impaired kidney function and shortened average treatment duration by 1.5–3.0 days without compromising clinical outcomes. Nevertheless, variations in study design, outcome measures and the absence of standardized imaging–AMS protocols limit the overall comparability and generalizability of findings.

**Conclusions:**

This review demonstrates that chest imaging, particularly ultrasound, can play a critical role in antimicrobial stewardship for community-acquired pneumonia in elderly and multimorbid patients. By enabling more accurate differentiation of bacterial from viral pneumonia, imaging supports safer initiation of antibiotics, promotes earlier IV-to-oral switching and facilitates appropriate renal dose adjustments. Collectively, these interventions contribute to shorter treatment durations without compromising outcomes. However, the evidence base remains heterogeneous, and the absence of standardized imaging–AMS protocols limits broader implementation. Advancing this field will require robust randomized controlled trials, economic evaluations and integration of imaging into guideline-based stewardship pathways, emphasized by close collaboration between radiologists, doctors and pharmacists.
